# CD164 is a host factor for lymphocytic choriomeningitis virus entry

**DOI:** 10.1073/pnas.2119676119

**Published:** 2022-03-02

**Authors:** Mark J. G. Bakkers, Alex Moon-Walker, Rasmus Herlo, Vesna Brusic, Sarah Hulsey Stubbs, Kathryn M. Hastie, Erica Ollmann Saphire, Tomas L. Kirchhausen, Sean P. J. Whelan

**Affiliations:** ^a^Department of Microbiology, Harvard Medical School, Boston, MA 02115;; ^b^Program in Virology, Harvard Medical School, Boston, MA 02115;; ^c^Center for Infectious Disease and Vaccine Research, La Jolla Institute for Immunology, La Jolla, CA 92037;; ^d^Department of Molecular Microbiology, Washington University in St. Louis, St. Louis, MO 63110;; ^e^Department of Cell Biology, Harvard Medical School, Boston, MA 02115;; ^f^Department of Pediatrics, Harvard Medical School, Boston, MA 02115

**Keywords:** lymphocytic choriomeningitis virus, CD164, arenavirus, virus entry

## Abstract

Lymphocytic choriomeningitis virus (LCMV) is a rodent-borne zoonotic arenavirus that causes congenital abnormalities and can be fatal for transplant recipients. Using a genome-wide loss-of-function screen, we identify host factors required for LCMV entry into cells. We identify the lysosomal mucin CD164, glycosylation factors, the heparan sulfate biosynthesis machinery, and the known receptor alpha-dystroglycan (α-DG). Biochemical analysis revealed that the LCMV glycoprotein binds CD164 at acidic pH and requires a sialylated glycan at residue N104. We demonstrate that LCMV entry proceeds by the virus switching binding from heparan sulfate or α-DG at the plasma membrane to CD164 prior to membrane fusion, thus identifying additional potential targets for therapeutic intervention.

## Significance

Lymphocytic choriomeningitis virus (LCMV) is the prototypic arenavirus and has been utilized for decades as a model to understand the host immune response against viral infection. LCMV infection can lead to fatal meningitis in immunocompromised people and can lead to congenital birth defects and spontaneous abortion if acquired during pregnancy. Using a genetic screen, we uncover host factors involved in LCMV entry that were previously unknown and are candidate therapeutic targets to combat LCMV infection. This study expands our understanding of the entry pathway of LCMV, revealing that its glycoprotein switches from utilizing the known receptor α-DG and heparan sulfate at the plasma membrane to binding the lysosomal mucin CD164 at pH levels found in endolysosomal compartments, facilitating membrane fusion.

Arenaviruses are enveloped, bisegmented ambisense RNA viruses found in close association with specific rodent hosts (1). The viruses are present in two geographic serologically distinct groups, termed “Old World” and “New World” arenaviruses (*SI Appendix*, Fig. S1*A*). Representative members of both groups cause fatal hemorrhagic disease in humans, including Lassa fever virus (LASV), and LuJo virus (LUJV) in the Old World group and Machupo (MACV), Junín, Guanarito, and Sabiá in the New World group (2, 3). The prototypic arenavirus, lymphocytic choriomeningitis virus (LCMV), is associated with the common house mouse (*Mus musculus*) and has a worldwide distribution, with a 2 to 5% seroprevalence in humans (4–9). Most human LCMV infections are asymptomatic or mild but can be fatal in transplant recipients and cause spontaneous abortion or congenital malformation during fetal development (10–14). Studies of LCMV infection of mice have led to landmark discoveries in viral immunology including major histocompatibility complex restriction and T-cell exhaustion (15, 16).

LCMV infection is initiated through binding of its attachment glycoprotein (GP) to the cell surface followed by virus internalization and subsequent fusion of viral with host-endolysosomal membranes (17–19). The GP is synthesized as a precursor polyprotein (GPC) that undergoes two proteolytic cleavage events during maturation by signal peptidase in the endoplasmic reticulum and by site-1 protease (S1P) in the Golgi apparatus (20–22). The N-terminal domain GP1 mediates receptor binding, while the carboxyl-terminal domain GP2 mediates membrane fusion. A stable signal peptide remains associated with the mature GP complex and plays roles in both glycoprotein trafficking and pH sensing through pH-sensitive interactions with GP2 that partially modulate the prefusion conformation at acidic pH (23–26). Membrane fusion is triggered by acidic pH, which is encountered in the endolysosomal compartment (19).

The cell-surface receptor for LASV and LCMV was reported as α-dystroglycan (α-DG) (27–29). The function of α-DG in virus infection depends on its glycosylation, which is catalyzed by the Golgi resident glycosyltransferase LARGE1 (28, 30, 31). Despite the importance of α-DG in infection, it alone is not sufficient for either LCMV or LASV infection. Iterative haploid genetic screens for host permissivity factors for the infection of cells by LASV uncovered additional host genes required for glycosylation of α-DG (32) and identified a key missing host cell receptor lysosomal associated membrane protein 1 (LAMP1) for infection (33). Biochemical evidence defined a pH-regulated receptor-switching mechanism for LASV entry whereby virus binds α-DG at neutral pH, but as endosomes become progressively acidified, GP dissociates from α-DG and binds LAMP1, leading to productive infection (33). Infection of cells by LCMV-Armstrong does not require α-DG (34) and does not require LAMP1 (33), indicating that there are additional unknown host factors that are involved in LCMV entry.

To identify the cellular factors that facilitate infection by LCMV-GP, we carried out a genome-wide CRISPR-Cas9 loss-of-function screen in the human lung epithelial cell line A549 using a vesicular stomatitis virus recombinant in which the native glycoprotein was replaced with that of LCMV (18). Perturbations of multiple host genes impeded infection including the lysosomal sialomucin, CD164. Through combined biochemical genetic and virological studies, we defined the central cysteine-rich domain (CRD) of CD164 and a specific, sialylated *N*-linked glycan within that domain required for infection. We detect direct binding of purified GP to CD164 at acidic pH and demonstrate that this interaction results in membrane fusion. This study identifies additional host factors in the entry pathway of LCMV into cells that may serves as targets for development of therapeutics to combat viral infection.

## Results

### Identification of LCMV Entry Factors through a Genome-Wide Screen.

**Fig. 1.**
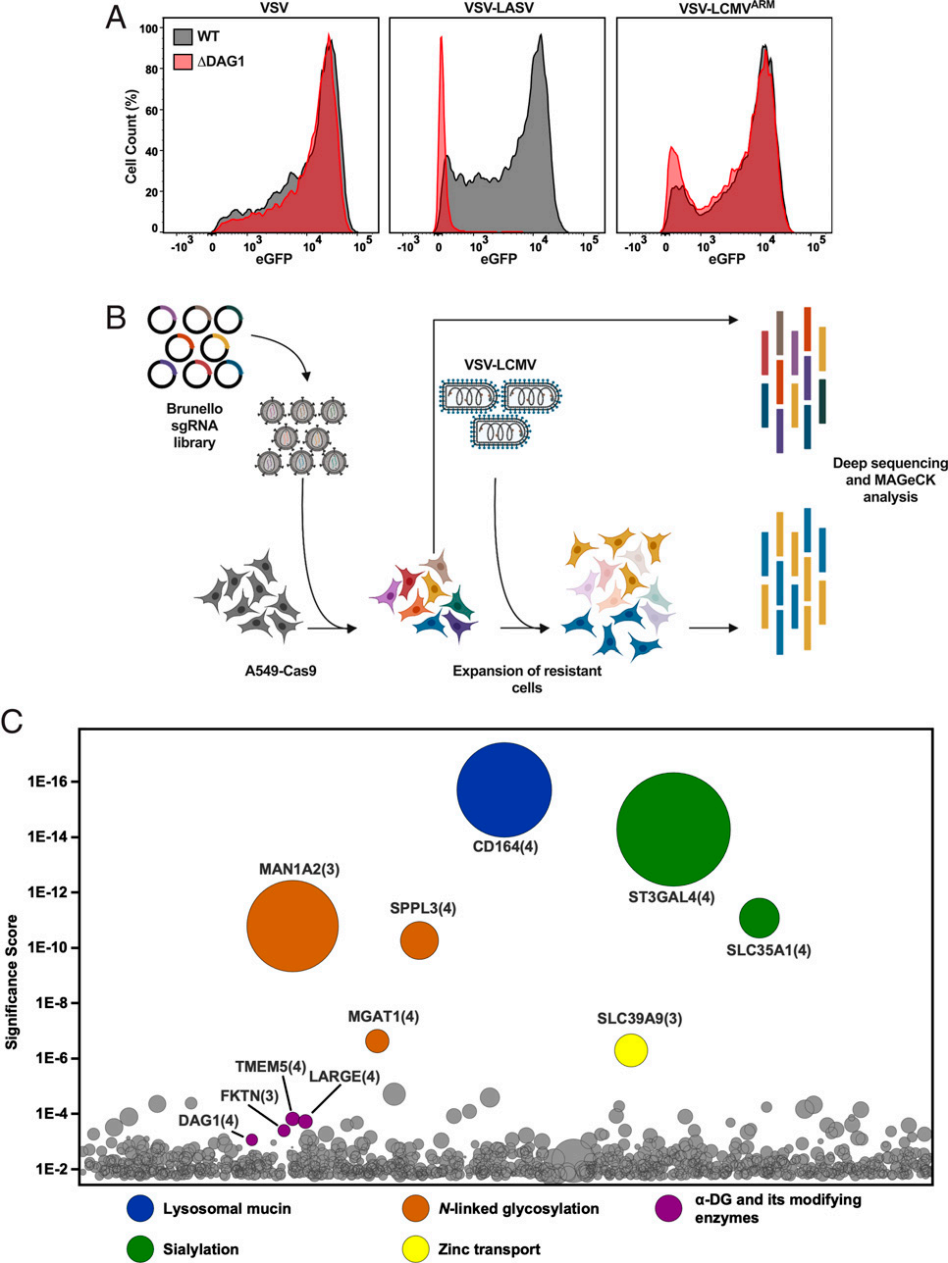
A genome-wide screen to identify host genes required for LCMV entry. (*A*) WT and ΔDAG1 A549 cells were infected with VSV, VSV-LASV, or VSV-LCMV at an MOI of 2. At 1 h postinfection, cells were washed to remove unbound virus and were subsequently replenished with fresh media. At 6 h postinfection, cells were fixed and subjected to flow cytometry to quantify infected cells by eGFP expression. Infectivity levels were normalized to WT A549 infectivity. A representative of three experimental replicates is shown. (*B*) Schematic of the CRISPR-Cas9 loss-of-function screen. Briefly, A549-Cas9 cells were transduced with the lentiviral Brunello sgRNA library and subjected to puromycin selection. The resulting mutagenized library was then infected with VSV-LCMV with a pool of the library taken to prior to use as a reference library. The resulting surviving cells were allowed to expand and then subjected to deep sequencing and MAGeCK analysis. (*C*) Results of the CRISPR-Cas9 genetic screen. The *y*-axis represents the significance score of each of the hits. The size of the circles represents the fold enrichment of each gene compared to the reference library. The number in parenthesis represents the number of guides that were enriched in the screen. Significant and relevant hits are grouped and colored by function.

To first validate that LCMV can infect cells independently of α-DG, we engineered the human lung epithelial cell line A549 by Cas9-mediated knockout (KO) of the DG gene *DAG1*. We verified that these cells lacked α-DG by flow cytometry (*SI Appendix*, Fig. S1*B*) and then monitored the effect on virus infection (*SI Appendix*, Fig. S1*C*). For infection studies, we used recombinant vesicular stomatitis virus (VSV) that expresses eGFP as a marker of infection and depends on its native glycoprotein or the GPC of LASV or LCMV for entry into cells and quantified the extent of infection at 6 h postinoculation by flow cytometry. As expected, VSV infection was unaffected in cells lacking α-DG (A549_ΔDAG1_), whereas VSV-LASV infection resulted in 5.1% infected cells and a reduction in the fluorescence intensity of eGFP (Fig. 1*A* and *SI Appendix*, Fig. S1*C*). Consistent with earlier findings that LCMV-Armstrong infects cells lacking α-DG (34), VSV-LCMV infection showed no significant reduction in both the number of infected cells and the fluorescence intensity in A549_ΔDAG1_ cells (Fig. 1*A* and *SI Appendix*, Fig. S1*C*). This result supports that LCMV-GP can mediate infection independent of α-DG.

We carried out a genome-wide loss-of-function screen in wild-type (WT) A549 cells that express α-DG to identify host genes that contribute to LCMV-GP–mediated entry (Fig. 1*B*, *SI Appendix*, Fig. S1*B*, and Dataset S1). Briefly, A549 cells stably expressing Cas9 (A549-Cas9) cells were transduced at low multiplicity of infection (MOI) with the Brunello lentiviral library that contains 77,441 single guide RNAs (sgRNAs) and encodes puromycin resistance (35). After 1 wk of puromycin selection, cells were infected with VSV-LCMV. Those that survived infection were expanded for 3 wk prior to isolation of genomic DNA for sequencing to identify the inactivating sgRNAs. We compared the reads for sgRNAs to their abundance in an unselected reference dataset using the model-based analysis of genome-wide CRISPR-Cas9 knockout (MAGeCK) software pipeline and plotted the genes targeted on a bubble plot according to their enrichment (Fig. 1*C*).

The most enriched gene in the screen, *CD164*, is expressed in most cell types and plays roles in cell adhesion, migration, proliferation, and muscle formation (36–38) (Dataset S1). CD164, also known as endolyn or MUC24, is a highly glycosylated, 197-amino-acid, type-I transmembrane sialomucin with a carboxyl-terminal NYxxL motif that targets the protein to the endolysosomal compartment (39). Additional highly enriched genes identified in the screen are involved in sialylation (*SLC35A1* and *ST3GAL4*), or *N*-linked glycosylation (*MAN1A2*, *SPPL3*, and *MGAT1*) (Dataset S1). Solute carrier (SLC35A1) performs an essential step in the biosynthesis of sialylated glycoconjugates by transporting CMP-activated sialic acid into the Golgi, where it serves as a substrate for linkage-specific sialyltransferases (40). *ST3GAL4* encodes a sialyltransferase that transfers α-2,3 sialic acid to galactose (41). All of these genes were enriched above *DAG1* and genes involved in the glycosylation of α-DG (Fig. 1*C* and Dataset S1).

### Identified Host Factors Affect Infection by LCMV and Closely Related Arenaviruses.

**Fig. 2.**
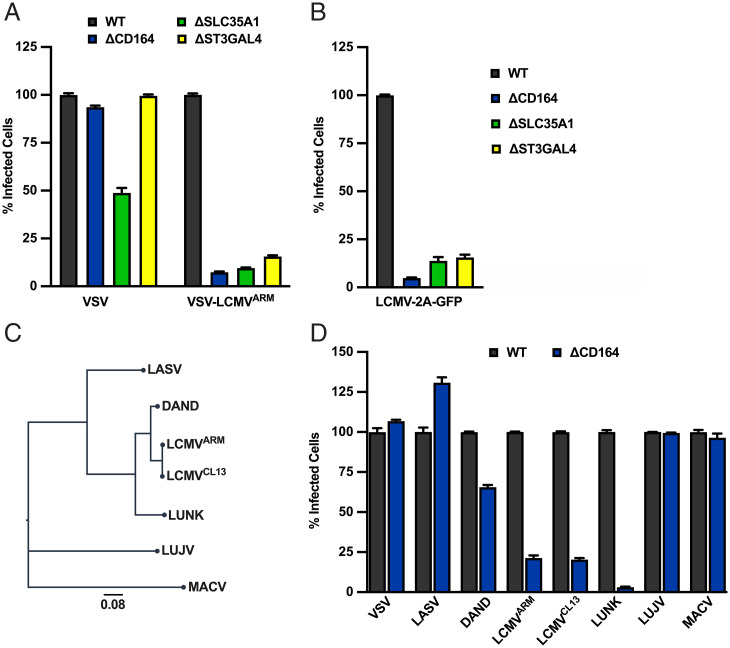
CRISPR-Cas9 significant hits are important LCMV entry factors. (*A*) WT, ΔCD164, ΔSLC35A1, and ΔST3GAL4 A549 cells were infected with VSV or VSV-LCMV at an MOI of 2. At 1 h postinfection, cells were washed to remove unbound virus and incubated for 6 h following infection. Cells were then fixed and subjected to flow cytometry to quantify infected cells by eGFP expression. Infectivity levels were normalized to WT A549 infectivity (*n* = 3 experimental replicates). (*B*) WT, ΔCD164, ΔSLC35A1, and ΔST3GAL4 A549 cells were infected with LCMV-2A-GFP at an MOI of 2. At 1 h postinfection, cells were washed to remove unbound virus and incubated for 24 h following infection. Cells were then fixed and subjected to flow cytometry to quantify infected cells by eGFP expression. Infectivity levels were normalized to WT A549 infectivity (*n* = 3 experimental replicates). (*C*) Phylogenetic tree based on GPC sequences of selected representative arenaviruses used in Fig. 2*D* using MEGA sequence alignment software. The scale bar indicates the number of amino acid substitutions per position. (*D*) WT and ΔCD164 A549 cells were infected with VSV or VSV recombinants bearing the representative arenavirus GPC at an MOI of 2. At 1 h postinfection, cells were washed, medium was added, and the cells were incubated for 6 h prior to flow cytometric analysis as described in the legend for Fig. 2*A*. Infectivity levels were normalized to WT A549 infectivity (*n* = 3 experimental replicates).

To validate the roles of CD164, SLC35A1, and ST3GAL4 in VSV-LCMV infection, we separately inactivated each gene in A549 cells using distinct guide RNAs (gRNAs) to those used in the Brunello library. We confirmed that the respective genes were inactivated in A549_ΔCD164_, A549_ΔSLC35A1_, A549_ΔST3GAL4_ by immunofluorescence microscopy and genomic DNA analysis (*SI Appendix*, Fig. S2 *A* and *B*) and verified the infection phenotype (Fig. 2*A* and *SI Appendix*, Fig. S2*C*). Each of the cell types were infected with either VSV-eGFP or VSV-eGFP-LCMV, and the extent of infection was determined by flow cytometry at 6 h postinfection (Fig. 2*A*). Infection of VSV-eGFP was unaffected in A549_ΔCD164_ and A549_ΔST3GAL4_ and showed a reproducible 50% reduction in A549_ΔSLC35A1_ as judged by the number of infected cells and their fluorescence intensity (Fig. 2*A* and *SI Appendix*, Fig. S2*C*). By contrast, VSV-LCMV infection showed a 84 to 92% reduction in each of the cell types as judged by the percentage of infected cells and their fluorescence intensity (Fig. 2*A* and *SI Appendix*, Fig. S2*C*). Infection of A549 cells with a recombinant LCMV-Armstrong engineered to express GFP, LCMV-2A-GFP, also show a marked reduction in infection upon inactivation of CD164, ST3GAL4, or SLC35A1 (Fig. 2*B* and *SI Appendix*, Fig. S2*D*). Collectively, these data directly demonstrate a role of CD164, SLC35A1, and ST3GAL4 in LCMV-GP–mediated infection at the entry step and validate their importance for infection with WT LCMV.

To examine whether loss of CD164 affected infection of cells mediated by other arenavirus GPs, we infected A549_ΔCD164_ cells with a panel of VSV-arenavirus chimeras (Fig. 2 *C* and *D* and *SI Appendix*, Fig. S2*E*). Infection of cells with VSV, VSV-LASV, VSV-LUJO, and VSV-MACV were unaffected by genetic ablation of CD164, whereas VSV-LCMV-Armstrong and the closely related GP Clone-13 (4) were inhibited (Fig. 2*D* and *SI Appendix*, Fig. S2*E*). To determine whether other LCMV-like viruses were affected by the loss of CD164, we generated VSV chimeras containing the GPs of Dandenong (DAND) virus isolate from a human transplant recipient (42) and LUNK virus from a rodent host (43). Infection of VSV-DAND and VSV-LUNK were reduced in A549_ΔCD164_ cells (Fig. 2*D* and *SI Appendix*, Fig. S2*E*), thus demonstrating that CD164 is a host factor specific to LCMV-related viruses but not other pathogenic human arenaviruses.

### Effect of CD164 Domain Deletions on LCMV Infection.

**Fig. 3.**
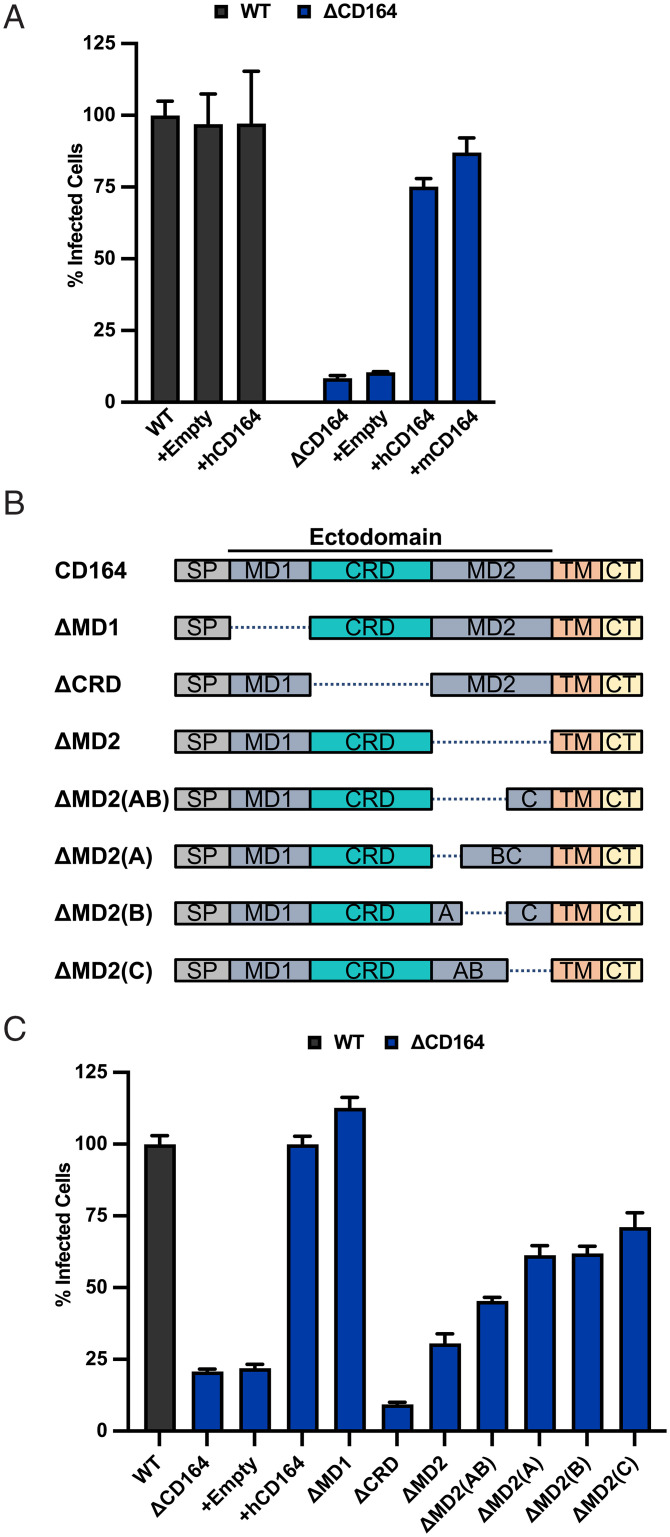
The CRD of CD164 is critical for its function as a host factor for LCMV. (*A*) WT and ΔCD164 HeLa cells were transduced by lentiviral vectors express null, human CD164, or mouse CD164. Cells were infected at an MOI of 2 with VSV-LCMV. At 1 h postinfection, cells were washed, medium was added, and the cells were incubated for 6 h prior to flow cytometric analysis as above. Infectivity levels were normalized to WT HeLa infectivity (*n* = 3 experimental replicates). (*B*) Truncation mutants used in infectivity studies within Fig. 3*C*. (*C*) WT, ΔCD164, and ΔCD164 HeLa cells expressing CD164 truncation mutants by lentiviral transduction were infected with VSV-LCMV at an MOI of 2. At 1 h postinfection, cells were washed, medium was added, and the cells were incubated for 6 h prior to flow cytometric analysis as above. Infectivity levels were normalized to WT HeLa infectivity (*n* = 3 experimental replicates).

To examine the requirements within CD164 for LCMV infection, we inactivated the gene in HeLa cells to facilitate genetic add-back studies. Using CRISPR-Cas9 and gRNAs, we inactivated CD164 in HeLa cells to generate HeLa_ΔCD164_ (*SI Appendix*, Fig. S3*A*). As with A549 cells, inactivation of CD164 inhibits infection by VSV-LCMV (Fig. 3*A* and *SI Appendix*, Fig. S3*B*). Infection of HeLa_ΔCD164_ cells was restored upon lentiviral complementation with human or mouse CD164 (Fig. 3*A* and *SI Appendix*, Fig. S3*B*), verifying that CD164 is specifically inactivated in cells and that mouse and human CD164 facilitate infection.

We next determined which domain(s) of CD164 are required for LCMV infection. CD164 is predicted to have two O-glycan–rich mucin domains (MD1 and MD2) separated by a CRD containing four *N*-linked glycosylation sites but no O-linked glycosylation (39). Guided by those predicted domain boundaries, we performed genetic add-back experiments in HeLa_ΔCD164_ cells with variants lacking the first mucin domain (ΔMD1), the CRD (ΔCRD), or the second mucin domain (ΔMD2) (Fig. 3*B*). Infection of VSV-LCMV was sensitive to loss of the CRD, partially sensitive to loss of MD2, and unaffected by loss of MD1 (Fig. 3*C* and *SI Appendix*, Fig. S3*C*). This result demonstrates the importance of the CRD and suggests that, like other mucins, MD2 may form a thin, stalk-like structure that extends the CRD domain a specific distance away from the membrane (44, 45). To test this hypothesis, we generated different versions of CD164 in which the three exons that make up MD2 (A, B, and C) were deleted individually or in pairs (Fig. 3*B*). Lentiviral vector complementation restored infection (Fig. 3*C* and *SI Appendix*, Fig. S3*C*), providing experimental support that the overall size or shape of MD2 contributes to the role of CD164 in LCMV infection.

### Effect of CD164 Glycosylation on LCMV Infection.

**Fig. 4.**
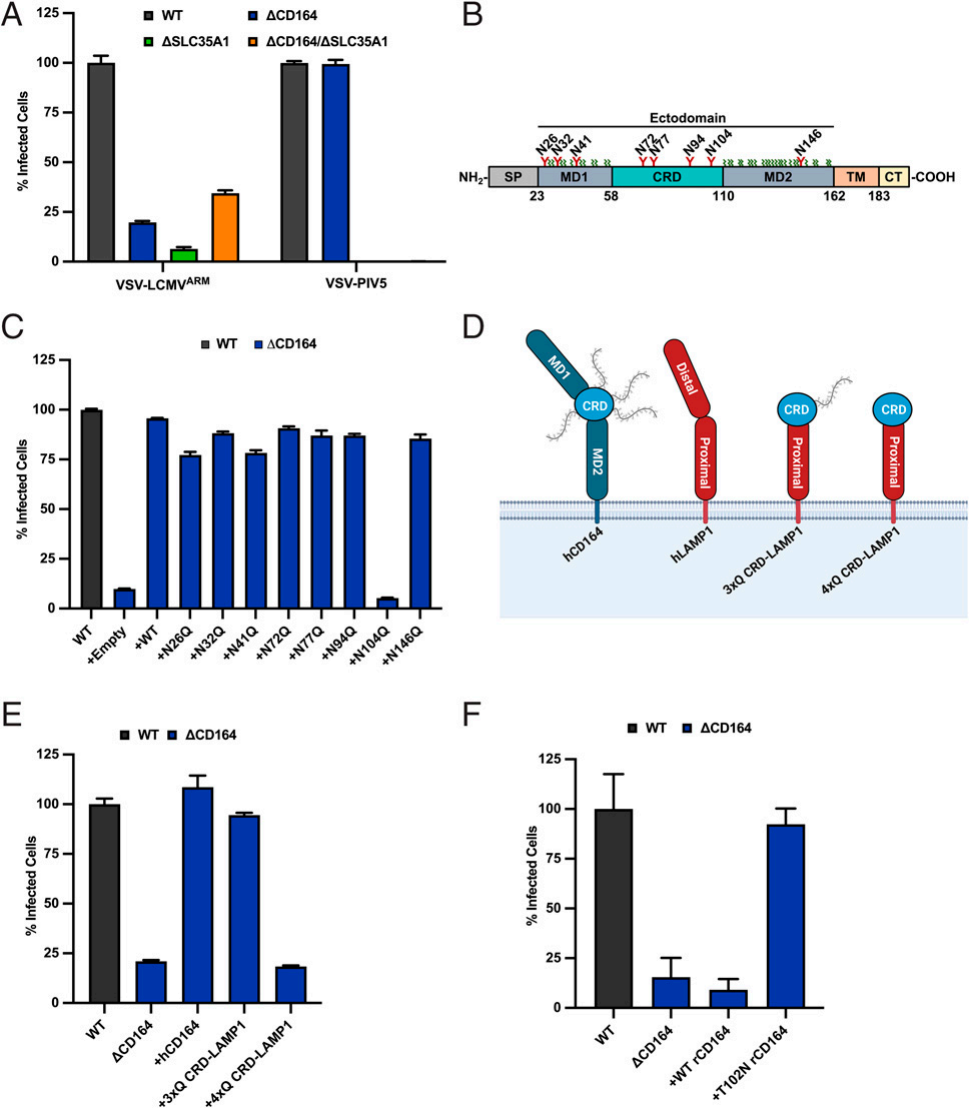
Effect of CD164 glycosylation on LCMV infection. (*A*) WT, ΔCD164, ΔSLC35A1, and ΔCD164/ΔSLC35A1 HeLa cells were infected with VSV-LCMV or VSV-PIV5 at an MOI of 2. At 1 h postinfection, cells were washed, medium was added, and the cells were incubated for 6 h prior to flow cytometric analysis as above. Infectivity levels were normalized to WT HeLa infectivity (*n* = 3 experimental replicates). (*B*) Topology of CD164. Predicted *N*-linked glycans are depicted in red, and predicted O-linked glycans are depicted in green. SP = signal peptide; TM = transmembrane Domain; CT = cytoplasmic tail. (*C*) WT, ΔCD164, and ΔCD164 HeLa cells expressing CD164 *N*-linked glycan mutants were infected with VSV-LCMV at an MOI of 2. At 1 h postinfection, cells were washed, medium was added, and the cells were incubated for 6 h prior to flow cytometric analysis as above. Infectivity levels were normalized to WT HeLa infectivity (*n* = 3 experimental replicates). (*D*) Schematic of CD164 CRD-LAMP1 chimeras used for infectivity studies within Fig. 3*E*. (*E*) WT, ΔCD164, and ΔCD164 HeLa cells expressing CD164 or the CD164 CRD-LAMP1 chimeras by lentiviral transduction were infected with VSV-LCMV at an MOI of 2. At 1 h postinfection, cells were washed, medium was added, and the cells were incubated for 6 h prior to flow cytometric analysis as above. Infectivity levels were normalized to WT HeLa infectivity (*n* = 3 experimental replicates). (*F*) WT, ΔCD164, and ΔCD164 HeLa cells expressing rCD164 or T102 rCD164 by lentiviral transduction were infected with VSV-LCMV at an MOI of 2. At 1 h postinfection, cells were washed, medium was added, and the cells incubated for 6 h prior to flow cytometric analysis as above. Infectivity levels were normalized to WT HeLa infectivity (*n* = 4 experimental replicates).

The genome-wide screen also identified genes involved in *N*-linked glycosylation and sialylation, including the CMP-sialic acid transporter SLC35A1 (Fig. 1*C* and Dataset S1). Inactivation of SLC35A1 in HeLa cells inhibits infection by VSV-LCMV and VSV expressing the glycoproteins of PIV5 (VSV-PIV5), a paramyxovirus that uses sialic acid as entry receptor (46) (Fig. 4*A* and *SI Appendix*, Fig. S4 *A* and *B*). Infection of HeLa_ΔSLC35A1_ and HeLa_ΔCD164/ΔSLC35A1_ cells by VSV-LCMV was diminished to a similar extent as in HeLa_ΔCD164_ cells (Fig. 4*A* and *SI Appendix*, Fig. S4*B*). This suggests that sialic acid plays a role in LCMV entry that is linked to CD164. We therefore examined whether sialylated glycosylation of CD164 is required for its function in LCMV infection by mutation of potential sites of *N*-linked glycosylation in CD164. We individually ablated each of the eight predicted *N*-linked glycosylation sites in the ectodomain of CD164 by mutation of the NxS/T motif to QxS/T and expressed those variants in HeLa_ΔCD164_ cells (Fig. 4*B*). All of the variants were expressed to similar levels to WT CD164, and with the exception of substitution N104Q, all restored infection (Fig. 4*C* and *SI Appendix*, Fig. S4 *C* and *D*).

To examine further the function of residue N104 in LCMV infection, we transduced HeLa_ΔCD164_ cells with a lentivirus expressing a chimeric CD164 in which we fused the CRD to the proximal domain of the lysosomal protein LAMP1 and mutated the predicted glycosylation sites at N72, N77, and N94 to glutamine (3xQ CRD-LAMP1) (Fig. 4*D*). This CRD-LAMP1 chimeric construct restored VSV-LCMV infection in HeLa_ΔCD164_ cells (Fig. 4*E* and *SI Appendix*, Fig. S4*E*). However, ablation of all four potential sites of *N*-glycosylation within the CRD of the chimeric protein, including N104 (4xQ CRD-LAMP1), inhibited infection (Fig. 4 *D* and *E* and *SI Appendix*, Fig. S4*E*).

The predicted glycosylation site at residue N104 is conserved across most mammalian species, except for the brown rat (*Rattus norvegicus*) and the Mongolian gerbil (*Meriones unguiculatus*) (*SI Appendix*, Fig. S5*A*). It is reported that, while neonatal brown rats can be infected by LCMV upon intracranial inoculation, adult rats appear resistant to infection as evident from serological studies and RT-PCR analysis rats cohoused with LCMV-infected mice (6, 47–50). To examine whether rat CD164 can functionally replace human CD164 for LCMV infection, we expressed in HeLa_ΔCD164_ cells, either WT rat CD164 (rCD164) or a mutant of rat CD164 in which the NxS/T sequence was restored (T102N rCD164). Expression of WT rat CD164 failed to increase VSV-LCMV infectivity (Fig. 4*F* and *SI Appendix*, Fig. S5*B*), but T102N rCD164 restored VSV-LCMV infectivity levels comparable to those observed in WT HeLas (Fig. 4*F* and *SI Appendix*, Fig. S5*B*). Collectively, these results support that the CRD of CD164 facilitates LCMV infection and that N104 is necessary for this activity.

### CD164 Is a Binding Partner of LCMV-GP.

**Fig. 5.**
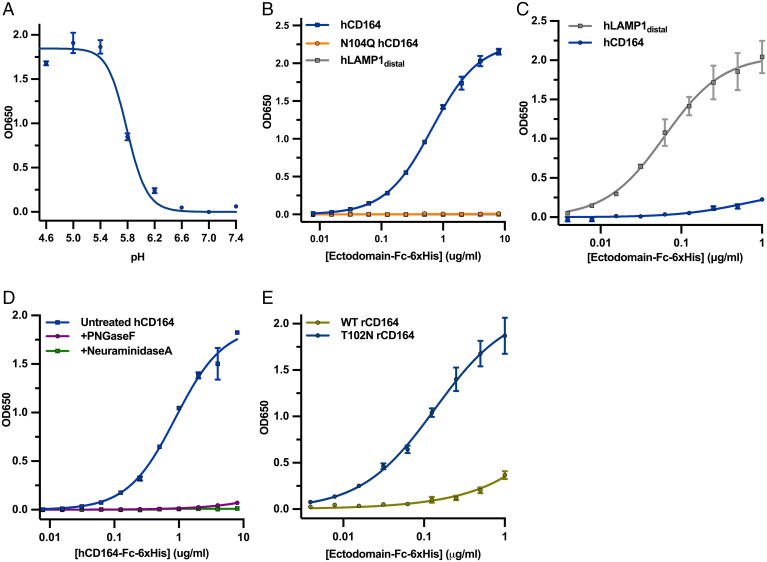
CD164 is a binding partner of LCMV GP. (*A*) Soluble LCMV-GP was coated onto 96-well, high-protein binding plates. Wells were washed, blocked, and then incubated with soluble CD164-Fc at the respective pH. Wells were washed extensively then incubated with goat anti-human IgG-HRP at the respective pH. Wells were extensively washed, and then TMB substrate was added, followed by a 650-nm stop solution, upon which the plate was read by a spectrophotometer plate-reader. The experiment was performed twice in duplicate, and a representative ELISA experiment is shown. (*B*) Soluble LCMV-GP was coated onto 96-half-well, high-protein binding plates. Wells were washed, blocked, and then incubated with soluble WT or N104Q CD164-Fc or LAMP1distal-Fc at pH 5. Wells were washed extensively and then incubated with goat anti-human IgG-HRP at pH 5. Wells were extensively washed, and then TMB substrate was added, followed by a 650-nm stop solution, upon which the plate was read by a spectrophotometer plate-reader. The experiment was performed twice in duplicate, and a representative ELISA experiment is shown. (*C*) Soluble LASV-GP was coated onto 96-well, high-protein binding plates. Wells were washed, blocked, and then incubated with soluble CD164-Fc or LAMP1Distal-Fc at pH 5. Wells were washed extensively and then incubated with goat anti-human IgG-HRP at pH 5. Wells were extensively washed, and then TMB substrate was added, followed by a 650-nm stop solution, upon which the plate was read by a spectrophotometer plate-reader. The experiment was performed twice in duplicate, and a representative ELISA experiment is shown. (*D*) Soluble LCMV-GP was coated onto 96-half-well, high-protein binding plates. Wells were washed, blocked, and then incubated with soluble untreated CD164-Fc or CD164-Fc that was treated either with PNGaseF (New England Biolabs) or NeuraminidaseA (New England Biolabs) at pH 5. Wells were washed extensively and then incubated with goat anti-human IgG-HRP at pH 5. Wells were extensively washed, and then TMB substrate was added, followed by a 650-nm stop solution, upon which the plate was read by a spectrophotometer plate-reader. The experiment was performed twice in duplicate, and a representative ELISA experiment is shown. (*E*) Soluble LCMV-GP was coated onto 96-well, high-protein binding plates. Wells were washed, blocked, and then incubated with soluble WT or T102N rCD164-Fc at pH 5. Wells were washed extensively and then incubated with goat anti-human IgG-HRP at pH 5. Wells were extensively washed, and then TMB substrate was added, followed by a 650-nm stop solution, upon which the plate was read by a spectrophotometer plate-reader. The experiment was performed twice in duplicate, and a representative ELISA experiment is shown.

To evaluate whether LCMV-GP interacts directly with CD164, we performed an enzyme-linked immunosorbent assay (ELISA) with purified recombinant LCMV-Armstrong GP ectodomain (LCMV_ARM_ sGP) and purified human WT CD164 ectodomain fused to the fragment crystallizable (Fc) region of human Immunoglobulin G (IgG) (hCD164) or the N104Q glycan mutant (N104Q hCD164) (*SI Appendix*, Fig. S5*C*). Soluble WT hCD164 bound to LCMV sGP in a pH-dependent manner with a threshold of pH <6.2 and maximal binding at pH <5.4, consistent with the low pH of late endosome/lysosomal compartments (Fig. 5*A*). At pH 5.0, neither N104Q hCD164 nor the soluble domain of LAMP1 that recognizes LASV-GP (hLAMP1distal) bound LCMV_ARM_ sGP (Fig. 5*B* and *SI Appendix*, Fig. S5*C*). Soluble LASV-GP ectodomain (LASV sGP) bound hLAMP1distal at pH 5.0, as expected, but hCD164 did not bind to LASV sGP under the same conditions (Fig. 5*C*). These data demonstrate that CD164 specifically interacts with LCMV-GP at pH <6.2 and that this interaction is dependent on an N residue at position 104. Treatment of purified recombinant hCD164 with the endoglycosidase PNGase F, which removes *N*-linked glycans or with NeuraminidaseA, which removes sialic acid from *N*-linked glycans, abrogated binding to LCMV-GP at pH 5.0 (Fig. 5*D* and *SI Appendix*, Fig. S5*D*). We also found that soluble T102N rCD164 bound LCMV sGP, whereas soluble WT rCD164 failed to bind (Fig. 5*E* and *SI Appendix*, Fig. S5*E*), further substantiating the infectivity results (Fig. 4*F* and *SI Appendix*, Fig. S5*B*). This result demonstrates that *N*-linked glycosylation and sialylation at N104 of CD164 is required for binding to LCMV-GP and provides an explanation for the identification of genes involved in glycosylation and sialylation required for infection in our screen.

### Effect of CD164 Mutants on Membrane Fusion.

**Fig. 6.**
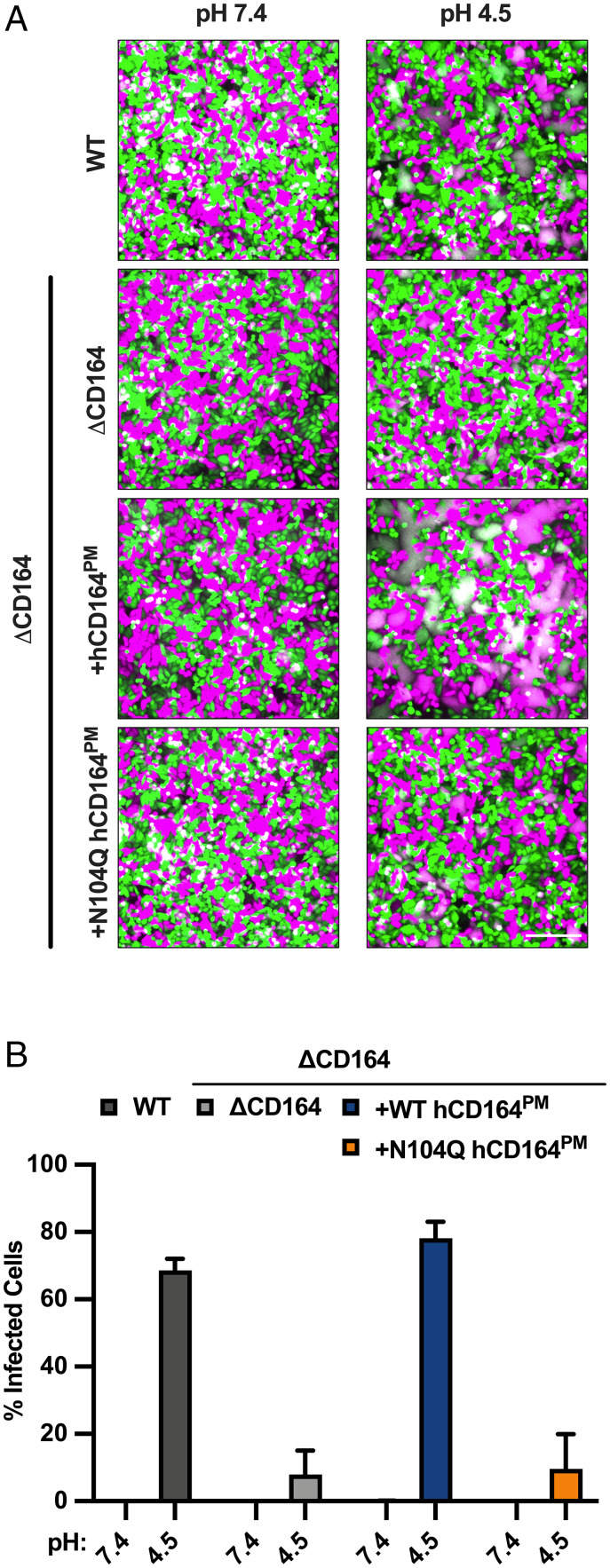
Effect of CD164 mutants on membrane fusion. (*A*) ΔCD164 HeLa cells expressing mCherry (magenta) by lentiviral transduction were designated as “effector” cells. WT, ΔCD164, or ΔCD164 expressing plasma membrane–localized CD164 mutants expressing eGFP (cyan) by lentiviral transduction were designated as “target” cells. Separately, effector and target cells were seeded on 6-well plates. Effector cells were transfected with pCAGGS LCMV-GP. At 4 h posttransfection, effector cells were washed. Both effector and target cells were trypsinized, resuspended, mixed, and seeded on poly-d-lysine–coated plates. At 24 h posttransfection, wells were imaged. Cells were washed, treated with media at pH 4.5 for 10 min, and then back neutralized with warm media. After 1 h, cells were imaged by fluorescence microscopy. A representative of three independent experiments is shown. (Scale bar, 500 µm.) (*B*) WT, ΔCD164, or ΔCD164 expressing plasma membrane–localized CD164 mutants were preincubated with 100 nM Bafilomycin A1 for 1 h. These cells were then infected with VSV-LCMV at an MOI of 30 on ice for 1 h. Cells were then washed to remove unbound virus and treated with media at pH 7.4 or 4.5 for 10 min, and then were back neutralized with media supplemented with 100 nM Bafilomycin A1. At 6 h post–acid pH treatment, cells were fixed and subjected to flow cytometry to quantify infected cells by eGFP expression (*n* = 3 experimental replicates).

Fig. 5 *A* and *B* demonstrates that LCMV-GP binds CD164 at acidic pH. The pH required for the interaction is consistent with that found in late endosomes/lysosomes, from which arenaviruses release their contents into the cytoplasm through GP-catalyzed membrane fusion (19). In the case of LASV, we previously found that the lysosomal resident protein LAMP1 promotes LASV-GP–mediated membrane fusion at acidic pH (33). We therefore posited that LCMV-GP binding CD164 could function in an analogous way to facilitate membrane fusion. To test this hypothesis, we designed a cell–cell fusion assay based on content mixing to determine whether surface expression of LCMV-GP in donor HeLa cells expressing mCherry could facilitate an acidic pH-dependent transfer into eGFP-expressing target HeLa cells expressing CD164 at the surface. Using this assay, we demonstrate that treatment of cells at pH 4.5 led to the formation of syncytia consistent with a fraction of CD164 present at the plasma membrane (51, 52) (Fig. 6*A* and *SI Appendix*, Fig. S6*A*). This pH-dependent syncytia formation was not detected in target cells lacking CD164 (Fig. 6*A* and *SI Appendix*, Fig. S6*A*). Target cells overexpressing a plasma membrane–targeted version of CD164 (hCD164_PM_) by ablation of the lysosomal targeting motif (51, 52) show enhanced syncytia formation upon exposure to acidic pH (Fig. 6*A*). We confirmed the surface expression of the plasma membrane–localized CD164 mutants by flow cytometry (*SI Appendix*, Fig. S6*A*). Consistent with the interaction of LCMV-GP depending on residue N104 within the CRD of CD164, target cells overexpressing a plasma membrane–targeted N104Q mutant failed to form syncytia (Fig. 6*A* and *SI Appendix*, Fig. S6*A*). We also included transfected VSV glycoprotein G (VSV-G) and an empty vector as positive and negative controls in this assay and used colocalization analysis to quantify syncytia formation (*SI Appendix*, Fig. S6*B*). Expression of VSV-G in effector cells readily formed syncytia in all four target cell types, while expression of the empty vector led to no visible syncytia in either of the target cells.

We next examined whether surface-expressed CD164 could facilitate virus–cell fusion. For this purpose, VSV-LCMV was bound to cells expressing the indicated version of CD164 at 4 °C, and membrane fusion was subsequently triggered by pulsing with acidic pH prior to warming. Successful fusion results in infection, which we monitored 6 h after acid pH treatment. Cells were incubated in the presence of Bafilomycin A1 both prior to binding of virus to cells and at the time of cell warming post–acid pH treatment to prevent fusion of any virions that were subsequently internalized. Consistent with the ability to mediate cell–cell fusion, cell-surface CD164 facilitates LCMV-GP–mediated infection upon exposure of surface-bound virus to pH 4.5 but not the N104Q CD164 mutant (Fig. 6*B* and *SI Appendix*, Fig. S6*C*).

### Heparan Sulfate Is a Cell-Surface Attachment Factor for LCMV.

**Fig. 7.**
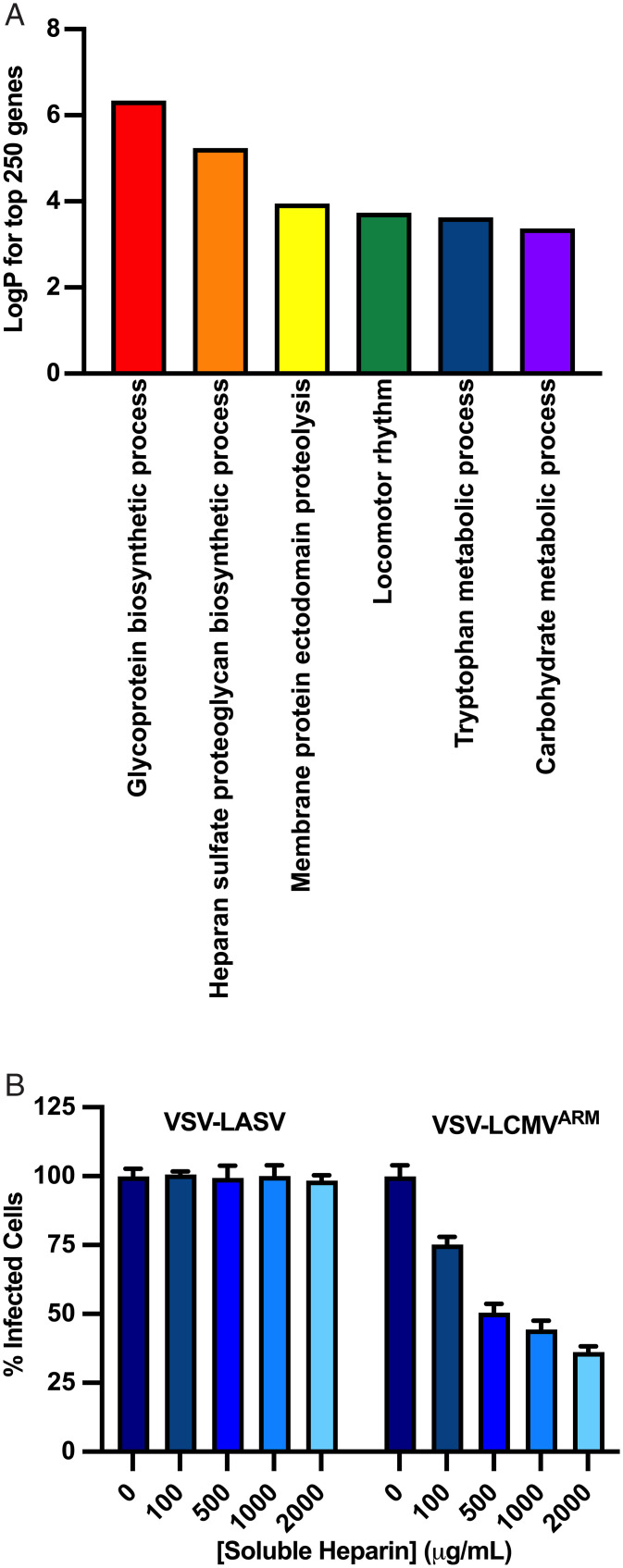
Heparan sulfate is an entry factor for LCMV. (*A*) Gene ontology analysis of the top 250 genes identified in the generic screen, showing the top seven pathways that were enriched. (*B*) VSV-LASV or VSV-LCMV at an MOI of 1 was incubated with soluble heparin at the indicated concentration for 45 min at room temperature. The medium on A549-Cas9 cells was aspirated and was replaced with the virus inoculum containing soluble heparin. At 1 h postinfection, cells were washed, fresh medium was added, and the cells were incubated for 6 h prior to flow cytometric analysis as described in *Materials and Methods*. Infectivity levels were normalized to 0 μg/mL soluble heparin. (*n* = 3 experimental replicates).

To determine how LCMV binds to the cell surface in the absence of α-DG, we further mined our screen data to look for possible attachment factors by performing pathway analysis on the top 250 genes using Metascape (Fig. 7*A*). The most significantly enriched pathways were GP and heparan-sulfate biosynthesis. Heparan sulfate is a ubiquitously expressed glycosaminoglycan that serves as an attachment factor for many viruses (53–57). To determine whether LCMV can also use heparan sulfate as an attachment factor, we monitored infection using VSV-LCMV in the presence of increasing concentrations of soluble heparin, a commercially available, highly sulfated analog of heparan sulfate. Incubation of VSV-LCMV, but not VSV-LASV, with soluble heparin inhibits infection of WT A549 cells in a concentration-dependent manner (Fig. 7*B* and *SI Appendix*, Fig. S7*A*), indicating that LCMV can also attach to cells through binding of heparan sulfate. A recent screen carried out with VSV-LCMV in human embryonic kidney 293 (HEK293) cells identified the importance of heparan sulfate in infection but did not identify CD164 (58). Although we do not know the basis for this distinction, this may reflect the fact that the screen was carried out in kidney cells (HEK293T) that were engineered to lack α-DG, not respiratory epithelial cells (A549) that contain α-DG. Our demonstration that purified CD164 engages in a specific interaction with LCMV-GP and not LASV-GP at acidic pH and that plasma membrane–targeted CD164 can lead to virus–cell fusion in response to acidic pH provide further support for its role in LCMV entry.

## Discussion

We report that the lysosomal mucin CD164 is a host factor that facilitates the entry of LCMV into host cells by binding to the viral GP at acidic pH and facilitating membrane fusion. As CD164 is predominantly located on lysosomal membranes (39, 59) and the interaction between CD164 and LCMV-GP requires acidic pH, this finding suggests that LCMV entry proceeds through a mechanism in which the viral GP engages α-DG at the plasma membrane and transitions to bind CD164 in the endolysosomal system. We also identified heparan sulfate as a previously unknown host factor that facilitates LCMV infection, suggesting that either α-DG or heparin sulfate can function to mediate the initial interactions of the virus at the plasma membrane. This finding offers an explanation for previous work in which LCMV infection does not require α-DG (34, 60).

A similar strategy, termed receptor switching, was previously reported for the related Old World arenavirus, LASV, which also binds α-DG at the cell surface and transitions to bind, at acidic pH, the lysosomal resident protein LAMP1 (33). For both LCMV and LASV, sialylation of the lysosomal host factor at a specific *N*-linked glycosylated residue by ST3GAL4 is required for infection, N104 in the case of CD164 and LCMV and N76 in the case of LAMP1 and LASV (33). Moreover, a further Old World arenavirus, LuJo, binds NRP2 at the plasma membrane and is dependent on an intracellular entry factor, CD63 (56, 61). Although the precise mechanism by which CD63 facilitates LUJV entry was not resolved, this finding suggests that cell entry of the Old World arenaviruses in general depends upon host factors that are resident on internal membranes of the endolysosomal system.

New World arenavirus GPs also require acidic pH to trigger membrane fusion and are also thought to initiate infection through fusion from within endolysosomal compartments (26, 62). Several New World arenaviruses employ transferrin receptor 1 (TfR1) to initiate infection (63, 64). Whether a similar pH-dependent transition facilitates engagement of an endolysosomal factor during New World arenavirus entry pathways has not been examined.

The location of a viral entry factor on endolysosomal membranes is also a shared mechanism for filovirus entry, in which, following engagement of one of many different attachment factors at the cell surface, endosomal proteolysis of the viral GP exposes the receptor binding site allowing engagement of the cholesterol transporter, Neimann-Pick C1 (65–69). This entry mechanism may offer the added benefit of shielding the receptor binding site on the GP from neutralizing antibodies that would thwart infection. For LCMV-GP, we did not precisely map the binding site for CD164, but the pH-dependent nature of the binding suggests that conformational changes in GP are required for CD164 binding in a manner analogous to LASV-GP engagement of LAMP1 (33). Structural studies will ultimately be required to precisely define how GP engages CD164 and identify the precise conformational transitions required for this interaction.

## Materials and Methods

### Cells and Viruses.

All cell lines used in this study [A549 (a gift from Nir Hacohen, Broad Institute of Massachusetts Institute of Technology and Harvard, Cambridge, MA), HeLa (a gift from Jan Carette, Department of Microbiology and Immunology, Stanford University School of Medicine, Stanford, CA), HEK293T (ATCC No. CRL-3216), Vero (ATCC No. CCL-81) and BSRT7 (70)] and any KO derivatives were cultured in Dulbecco’s modified Eagle’s medium (DMEM) supplemented with 5% fetal bovine serum (FBS) and penicillin–streptomycin–kanamycin and maintained at 37 °C and 5% CO_2_. Cell lines were routinely tested and found to be free of mycoplasma. Viruses were grown on either Vero (VSV-LASV, VSV-LUJV, and LCMV-2A-GFP) or BSRT7 cells (VSV, VSV-MACV, VSV-LCMV, VSV-DAND, and VSV-LUNK). All viruses were titrated by flow cytometry on A549 and HeLa cells. HEK-293T cells were used for the generation of recombinant lentiviruses and the production of Fc-tagged soluble CD164. ExpiCHO cells (Thermo Fisher Scientific No. A29127) were also used in the production of Fc-tagged soluble CD164.

### Generation of Gene-Edited Cells.

KO cells were generated by CRISPR-Cas9–mediated genome editing as described previously (35). In brief, cells were transfected with a plasmid-encoding Cas9, two sgRNAs targeting the gene of interest, and puromycin resistance (pCRISPR-2xgRNA-Puro, a kind gift from F. J. M. van Kuppeveld, Department of Biomolecular Health Sciences, Utrecht University, Utrecht, The Netherlands). Cells were transfected using Turbofectin 8.0, and transfected cells were selected with puromycin for 3 d. Single cell clones were isolated on a BD FACSAria, and KO was confirmed by diagnostic PCR and DNA sequencing. CD164 KO was further confirmed by immunofluorescence staining using polyclonal sheep IgG directed against human CD164’s extracellular domain (R&D Systems No. AF57910) and genomic DNA analysis. Loss of α-DG expression was further confirmed by flow cytometry using antibody IIH6 (Santa Cruz Biotechnology No. sc-53987). SLC35A1 and ST3GAL4 knockouts were confirmed by genomic DNA analysis.

### Genome-Wide CRISPR-Cas9 Screen.

To generate the sgRNA CRISPR KO library, 120 × 10^6^ A549-Cas9 cells (a kind gift from Nir Hacohen) were transduced with the Brunello lentiviral library (35) in the presence of polybrene at an MOI of 0.3 to reach an average coverage of 500-fold for each individual sgRNA. After 24 h, the cells were placed under selection of 10 μg/mL puromycin and passaged for 7 d. At that time, a reference dataset was collected by isolating genomic DNA from 40 × 10^6^ cell extractions using the Qiagen genomic DNA extraction kit according to the manufacturer’s protocol. On day 7 posttransduction, 100 × 10^6^ A459-Cas9 cells were inoculated with VSV-LCMV at an MOI of 0.2 for 1 h, after which the cells were washed and the medium was replaced. Infection was allowed to proceed for a period of 3 wk, with medium being replaced every 24 h. After 3 wk, resistant cells had grown out into colonies, which were collected for genomic DNA isolation. Next, PCR was performed to amplify the sgRNA cassettes from the transduced Brunello library lentivirus for both the reference and selected cells. In the PCR, sequencing adaptors and barcodes were introduced. For each sample, we performed 50 μL PCR reactions in eight replicates. The PCR reactions consisted of 3 μg DNA, Q5 enzyme, Q5 buffer, Q5 high GC enhancer, deoxyribose nucleotide triphosphates (dNTPs), uniquely barcoded P7 primer, P5 stagger primer mix, and water. The PCR cycling protocol entailed 5 min initial denaturation at 98 °C followed by 30 s at 98 °C, 30 s at 62 °C, and 30 s at 72  °C for 28 cycles and was concluded by a final 10-min extension at 72 °C. The replicate PCR reactions were pooled, and the gel was purified and sequenced on a MiSeq (Illumina). Enrichment of sgRNAs after VSV-LCMV selection as compared to the unselected reference dataset was calculated using the MAGeCK software pipeline.

### Generation of Recombinant VSV.

VSV recombinants expressing eGFP and the heterologous GPs of LCMVarm, LCMVcl-13, LASV, LUJV, MACV, and PIV5 have been generated previously (18, 32, 56, 57). VSV-DAND and VSV-LUNK were generated for this study using the deposited GPC sequences (GenBank identifiers: EU136038.1 and AB693150.1, respectively). Cloning and rescue of recombinant virus were performed using established methods (71, 72).

### Reconstitution of Protein Expression by Lentiviral Add-back.

To reconstitute protein expression in KO cells, we used a lentiviral delivery system. In brief, the complementary DNAs (cDNAs) of human CD164, mouse CD164, human SLC35A1, or human ST3GAL4 were purchased from the Harvard plasmid repository and cloned into the lentiviral cDNA expression plasmid pCW62-Puro (Harvard plasmid repository No. EvNO00438621). Distinct domain-deletion variants of human CD164, as well as *N*-linked glycosylation mutants, were generated using Q5 site-directed mutagenesis cloning (New England Biolabs). Recombinant lentiviruses were produced by Turbofectin 8.0–mediated transfection of HEK293T cells with pCW62, pCAGGS-VSV-G (73), and the packaging plasmid psPAX2 (a gift from Didier Trono, Department of Molecular and Cellular Biology, École Polytechnique Fédérale de Lausanne, Lausanne, Switzerland; Addgene plasmid No. 12260). After 36 h, cell supernatant was collected and filtered over a 0.45-µM filter prior to low MOI transduction of A549 or HeLa cells. A549 and HeLa cells were selected and maintained under 5 and 1 µg/mL blasticidin and puromycin, respectively.

### Recombinant Protein Design, Expression, and Purification.

The ectodomain of CD164, encompassing residues 1 through 162, was cloned into pCMVi-SV40i vector (Addgene plasmid No. 72098), which fuses the CD164 ectodomain to the Fc region of IgG to make pCMVi-hCD164-Fc-6xHis. The N104Q mutant was generated by Q5 site-directed mutagenesis cloning (New England Biolabs). LAMP1distal-Fc-6xHis was generated by cloning LAMP1 (residues 1 through 197) into the pCMVi-SV40i vector. Protein was purified from HEK293T cells via transient transfection of the desired pCMVi vector. A T150 flask confluent with HEK293T cells was transfected with 30 μg of the respective pCMVi plasmid using Turbofectin (Origene) according to the manufacturer’s protocol. At 24 h posttransfection, cells were washed, media was replaced with FreeStyle expression media (Thermo Fisher Scientific), and cells were incubated for 5 d at 34 °C. Supernatants were collected, filtered through a 0.45-μm filter, and mixed with Protein A beads that were washed in 1× phosphate buffered saline (PBS). The supernatant/Protein A slurry mix was allowed to rotate overnight at 4 °C. The supernatant/Protein A slurry mix was allowed to flow through gravity filtration columns. Protein was eluted from Protein A beads using 0.1 M citric acid, pH 3.0 and neutralized with equal volumes of 1 M Tris HCl buffer, pH 8.0. Eluted protein was then dialyzed overnight in a dialysis cassette (Slide-A-Lyzer 10K, Thermo Fisher Scientific No. 66382) overnight in PBS.

For recombinant Fc-tagged proteins used for Fig. 5 *B* and *D*, protein was expressed using the ExpiCHO Transfection System (Thermo Fisher Scientific), following the manufacturer’s protocol. The supernatant from the transfected ExpiCHO cells was purified by Protein A affinity chromatography (GE Healthcare No. 17549851), followed by size exclusion chromatography (SEC) purification over a S6 Increase column (GE Healthcare No. 29091596) in Tris-buffered saline (TBS) (25 mM Tris and 150 mM NaCl, pH 7.4).

For the deglycosylation and desialylation of soluble hCD164-Fc-6xHis, the protein was treated with either PNGaseF (New England Biolabs) or Neuraminidase A (New England Biolabs) according to the manufacturer’s protocol. The recombinant hCD164 protein was then buffer exchanged into TBS (25 mM Tris and 150 mM NaCl, pH 7.4).

### ELISA.

High protein-binding plates (Corning No. 9018) were coated with 500 ng/well soluble LCMV sGP or soluble LASV sGP (74, 75) resuspended in 1× PBS and incubated at 37 °C for 1 h. Wells were then incubated with a blocking buffer composed of 3% bovine serum albumin (BSA) in 1× PBS for 1 h at 37 °C for 1 h. Wells were extensively washed in ELISA buffer composed of 3% BSA and 0.05% Tween 20 in PBS^+Mg/+Ca^ at the respective pH. The indicated soluble Fc-tagged protein was resuspended in ELISA buffer in a twofold dilution series in ELISA buffer at the respective pH and incubated at 37 °C for 1 h. Wells were washed extensively in ELISA buffer and then incubated with a 1:500 dilution of goat α-human IgG conjugated to horseradish peroxidase (HRP) in ELISA buffer at the respective pH for 1 h at 37 °C. Wells were once again washed extensively with ELISA buffer at the respective pH. TMB (3,3′,5,5′-tetramethylbenzidine) ELISA Substrate (Highest Sensitivity) (Abcam No. ab171522) was added, followed by equal volumes of 650 nm Stop Solution for TMB Substrate (Abcam No. ab171531). The optical density at 650nm (OD650) of each well was read using a spectrophotometer plate reader (Biotek Synergy, HTX Multimode Reader).

For ELISAs performed in Fig. 5 *B* and *D*, half-area high protein-binding plates (Corning No. 07–200-37) were used instead and were coated with 200 ng/well of soluble LCMV sGP. The ELISA protocol for Fig. 5 *B* and *D* remains the same, except that the ELISA buffer used was composed of a 0.1 M citrate buffer, pH 5 (supplemented with 1 mM calcium chloride, 1 mM magnesium chloride, 3% BSA, and 0.05% Tween 20). The OD650 of each well was read using a spectrophotomer plate reader (Tecan, Spark Multimore Reader).

### Immunofluoresence Staining.

WT HeLa, HeLa_ΔCD164_, or HeLa_ΔCD164_ cells lentivirally transduced to express a recombinant WT (hCD164 PM) or N104Q (N104Q hCD164 PM), in which the NYxxL lysosomal targeting motif was deleted to increase plasma membrane (PM) expression to yield ΔCD164+hCD164 PM HeLa and ΔCD164+N104Q hCD164 PM HeLa, respectively, were used for immunofluoresence staining. The four different cell types were plated on 6-well plates, and 24 h later, cells were resuspended in PBS and 5 mM ethylenediamine tetraacetic acid. Cells were then washed twice in PBS and then fixed in 4% paraformaldehyde (PFA) for 20 min. Cells were washed twice on ice in PBS and then blocked in 1% BSA diluted in PBS (PBSA) for 30 min. Cells were then incubated on ice in a 1:500 dilution of sheep anti-human CD164 (R&D Systems No. AF5790) diluted in PBSA. Cells were washed three times in PBSA and then incubated with a 1:600 dilution of Donkey anti-sheep-488 (Thermo Fisher Scientific) in PBSA on ice. Cells were washed three times in PBSA and then subjected to flow cytometry analysis (FACS Canto), and the data were analyzed using the FlowJo software.

### Cell–Cell Fusion Assay.

WT HeLa, HeLa_ΔCD164_, or HeLa_ΔCD164_ cells lentivirally transduced to express a recombinant WT (hCD164^PM^) or N104Q hCD164 (N104Q hCD164^PM^), in which the NYxxL lysosomal targeting motif was deleted to increase cell-surface expression to yield ΔCD164+hCD164^PM^ HeLa and ΔCD164+N104Q hCD164^PM^ HeLa, respectively. WT, ΔCD164, ΔCD164+hCD164^PM^, and ΔCD164+N104Q hCD164^PM^ HeLa cells were then transduced with a lentivirus expressing eGFP and encoding the blasticidin resistance gene. This yielded WT+eGFP, ΔCD164+eGFP, ΔCD164+hCD164^PM^+eGFP, and ΔCD164+N104Q hCD164^PM^+eGFP HeLa cells. Additionally, ΔCD164 HeLa cells were transduced with a lentivirus encoding mCherry and expressing the puromycin resistance gene, yielding ΔCD164+mCherry HeLa cells. The ΔCD164+mCherry HeLa cells were transiently transfected with a plasmid-encoding VSV-G, LCMV-GPC, or an empty vector using Turbofectin (Origene) according to the manufacturer’s protocol. At 4 h posttransfection, cells were trypsinized and mixed with either WT+eGFP, ΔCD164+eGFP, or ΔCD164+CD164^PM^+eGFP and ΔCD164+N104Q CD164^PM^+eGFP HeLa cells and plated on poly-d-lysine–coated plates overnight at 37 °C. Cells were imaged before exposure to acidic buffer. Cells were then incubated with DMEM at pH 4.5 for 10 min at 37 °C and then neutralized with DMEM containing 2% FBS and 20 mM Hepes buffer. At 1 h after treatment with acidic media, cells were imaged to look for syncytia formation.

### Acid Bypass.

Approximately 1 × 10^5^ cells/well of WT HeLa, HeLa_ΔCD164_, HeLa_ΔCD164_+hCD164^PM^, and HeLa_ΔCD164_+N104Q hCD164^PM^ were plated in a 48-well plate. At 24 h, cells were pretreated with media supplemented with 100 nM Bafilomycin A1 (Sigma-Aldrich) for 1 h at 37 °C. Cells were placed on ice and then overlaid with VSV-LCMV-eGFP at an MOI of 30. Virus binding was then allowed to occur for 1 h. Cells were then incubated with warm DMEM (37 °C, pH 7.4 or 4.5) for 10 min, washed to remove unbound virus, and then incubated with media supplemented with 100 nM Bafilomycin A1 for 6 h. Cells were then trypsinized, fixed in 4% PFA, and then subjected to flow cytometry analysis (FACS Canto) for quantification of infection, and data were analyzed using FlowJo software.

### Recombinant VSV and LCMV Infections.

Cells were infected with the indicated eGFP-expressing VSV recombinant or LCMV-2A-GFP (76) at the indicated MOI for 1 h at 37 °C. Cells were then washed with Hank's balanced salt solution to remove unbound virus and replenished with fresh media. At 6 h postinfection (or 24 h for LCMV-2A-GFP), cells were trypsinized and fixed in 4% PFA and subjected to flow cytometry analysis (FACS Canto), and the data were analyzed using FlowJo software.

### Heparin Blocking.

Heparin (H3393, Sigma-Aldrich) was dissolved in PBS^+Ca/+Mg^ (Sigma-Aldrich). VSV-LASV and VSV-LCMV were incubated with the indicated concentration (100, 500, 1,000, or 2,000 µg/mL heparin) in PBS^+Ca/+Mg^ at an MOI of 1 for 45 min at 37 °C and then added to A549 cells. After 30 min at 37 °C, the virus inoculum was removed, and cells were washed twice. Infection was then allowed to progress for 5 h, after which cells were collected and fixed with 4% PFA and the percentage of infected cells was measured by flow cytometry (FACS Canto). Data analysis was performed using FlowJo software, and the values were normalized to mock treatment (0 µg/mL heparin).

### Confocal Microscopy.

A549 cells were seeded on EtOH-washed 12-mm glass coverslips at a density of 0.1E6 cells per well and incubated at 37 °C for 24 h. Medium was aspirated, and cells were washed twice with PBS^+Ca/+Mg^ prior to fixation with PBS and 2% PFA for 15 min at room temperature. Cells were permeabilized with 0.5% TritonX-100 diluted in PBS for 10 min. Cells were washed twice and blocked for 30 min with 2% BSA in PBS. Primary antibodies (anti-CD164, R&D Systems No. AF5790; anti-LAMP1, Millipore No. AB2971) were diluted according to the manufacturer’s recommendations and added for 1 h in PBS with 0.5% BSA. After incubation, coverslips were washed three times and then incubated with secondary antibody for 30 min. Covers slips were washed three more times and mounted using ProlongGold on glass slides. Images were acquired with an FV1000 confocal laser scanning microscope (Olympus) equipped with an oil immersion objective (U Plan S Apo 100×, numerical aperture 1.40) and Olympus Fluoview Software (3.0 a) and a Leica Microsystems microscope. Representative images were acquired with Leica confocal software (Leica Microsystems) at 60-fold magnification.

## Supplementary Material

Supplementary File

Supplementary File

## Data Availability

All study data are included in the article and/or supporting information.
