# Supplementary material for: CD164 is a host factor for lymphocytic choriomeningitis virus entry
Source: Proc Natl Acad Sci U S A. 2022 Mar 2;119(10):e2119676119. doi: 10.1073/pnas.2119676119 (PMC8915965; doi:10.1073/pnas.2119676119)
Supplement: Supplementary File [file pnas.2119676119.sapp.pdf]

PNAS
www.pnas.org

# Supplementary Information for
## CD164 is a host factor for lymphocytic choriomeningitis virus entry

**Authors:** Mark J. G. Bakkers[1,7,#], Alex Moon-Walker[1,2,5,6,#], Rasmus Herlo[3], Vesna Brusic[1], Sarah Hulsey Stubbs[1], Kathryn M. Hastie[5], Erica Ollmann Saphire[5], Tomas L. Kirchhausen[3,4] and Sean P. J. Whelan[1,6,*]

**Affiliations:**
[1]Department of Microbiology, Harvard Medical School; Boston MA 02115.
[2]Program in Virology, Harvard Medical School; Boston MA 02115.
[3]Department of Cell Biology, Harvard Medical School; Boston, MA 02115.
[4]Department of Pediatrics, Harvard Medical School; Boston, MA 02115.
[5]La Jolla Institute for Immunology; La Jolla, CA 92037.
[6]Department of Molecular Microbiology, Washington University in St. Louis; St Louis MO 63110
[7]Current address: Janssen Vaccines & Prevention BV; Leiden, The Netherlands
[#]Contributed equally
*Corresponding author and lead contact. Email: spjwhelan@wustl.edu. Phone: 314-286-1585. Address: MSC 8230-16-09240, 660 S. Euclid Avenue, St. Louis MO 63110.

**This document includes:**

Figures S1-S7

SI Appendix Figure Legends

**Other supplementary materials for this manuscript include the following:**

Dataset S1. CRISPR-Cas9 VSV-LCMV genetic screen data (attached as a separate Excel file).

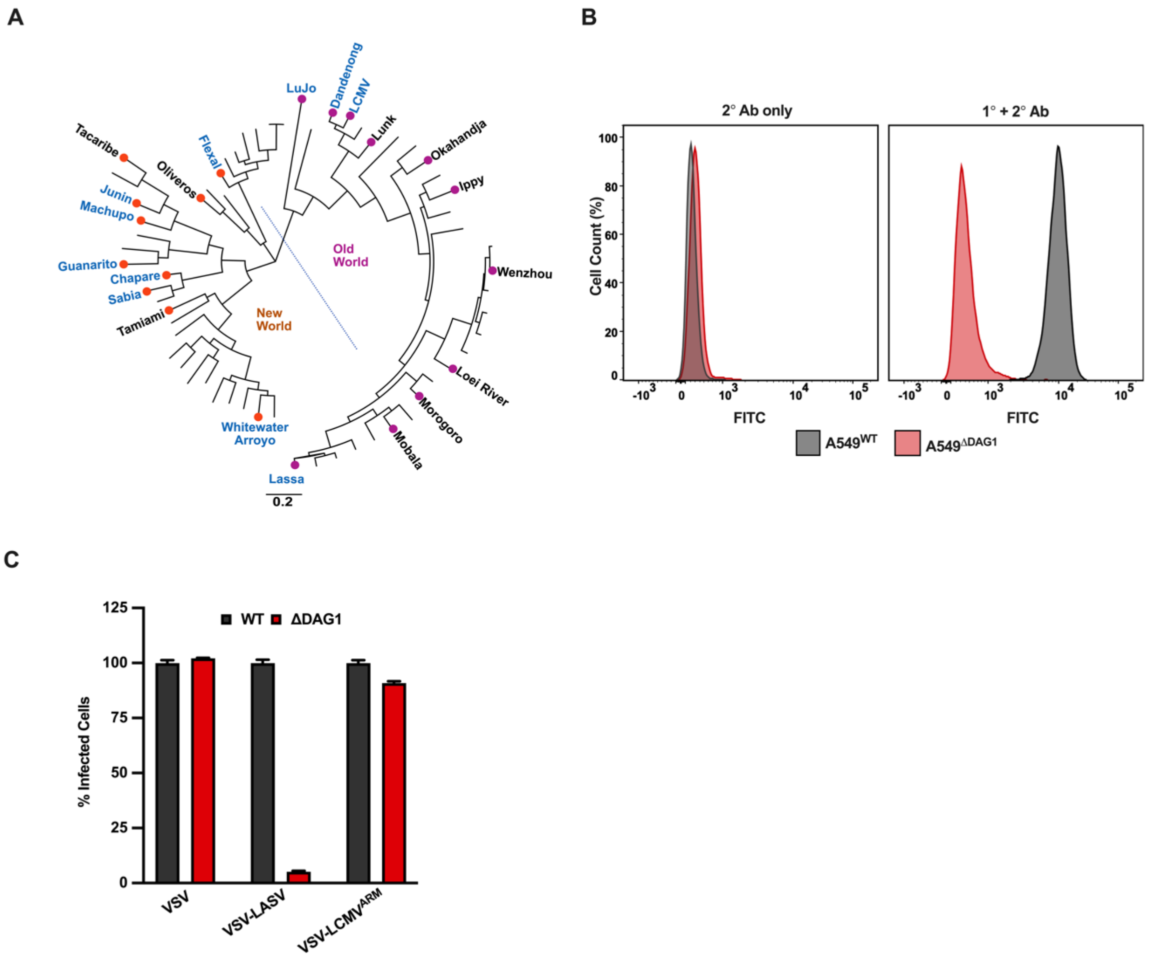
A
B
C
LuJo
Dandenong
LCMV
Lunk
Okahandja
Ippy
Wenzhou
Loei River
Morogoro
Mobala
Lassa
Tacaribe
Flexal
Oliveros
Junin
Machupo
Guanarito
Chapare
Sabia
Tamiami
Whitewater Arroyo
Old World
New World
0.2
2° Ab only
1° + 2° Ab
Cell Count (%)
FITC
A549WT
A549ΔDAG1
WT
ΔDAG1
% Infected Cells
VSV
VSV-LASV
VSV-LCMVARM

**Supplemental Figure 1. Related to Figure 1.**

A) Phylogenetic tree based on the GPC sequences on known mammarenavirus GPC amino acid sequences using MEGA sequence alignment software. In blue are mammarenaviruses that are known to infect humans. Scale bar indicates the amount of amino acid substitutions per position.

B) WT and **Δ**DAG1 A549 cells were cell-surface stained with anti alpha-dystroglycan antibody as well with as a secondary only control. These cells were subjected to flow cytometry analysis to determine the level of cell-surface expression of alpha-dystroglycan.

C) Infectivity data from Figure 1A normalized to WT A549. (n=3 experimental replicates).

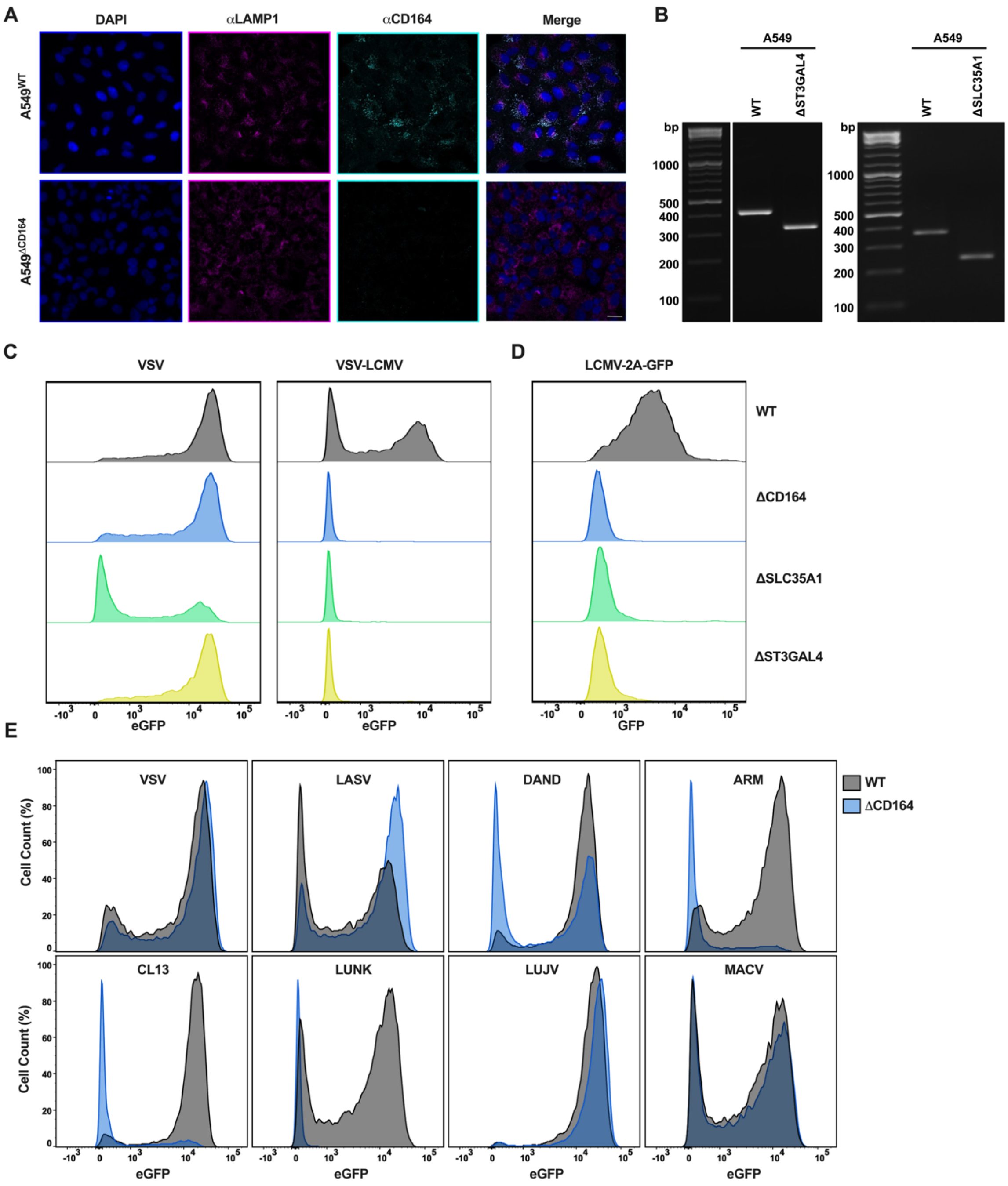
A
DAPI
αLAMP1
αCD164
Merge
A549WT
A549ΔCD164
B
A549
WT
ΔST3GAL4
bp
1000
500
400
300
200
100
A549
WT
ΔSLC35A1
C
VSV
VSV-LCMV
eGFP
D
LCMV-2A-GFP
WT
ΔCD164
ΔSLC35A1
ΔST3GAL4
GFP
E
VSV
LASV
DAND
ARM
CL13
LUNK
LUJV
MACV
WT
ΔCD164
Cell Count (%)
eGFP

**Supplemental Figure 2. Related to Figure 2.**

A) Fluorescence microscopy analysis of WT and **Δ**CD164 A549 cells. Briefly, cells were fixed, permeabilized, and stained with an anti-CD164 antibody and anti-LAMP1 antibody and DAPI. Cells were then imaged using confocal microscopy. Scale bar, 20 μm.

B) Qualitative PCR on parental A549 cells and the indicated gene KO cells. By combining two gRNA's an exon-intron boundary is removed, as confirmed by the smaller band size.

C) Representative flow cytometry data from Figure 2A.

D) Representative flow cytometry data from Figure 2B.

E) Representative flow cytometry data from Figure 2D.

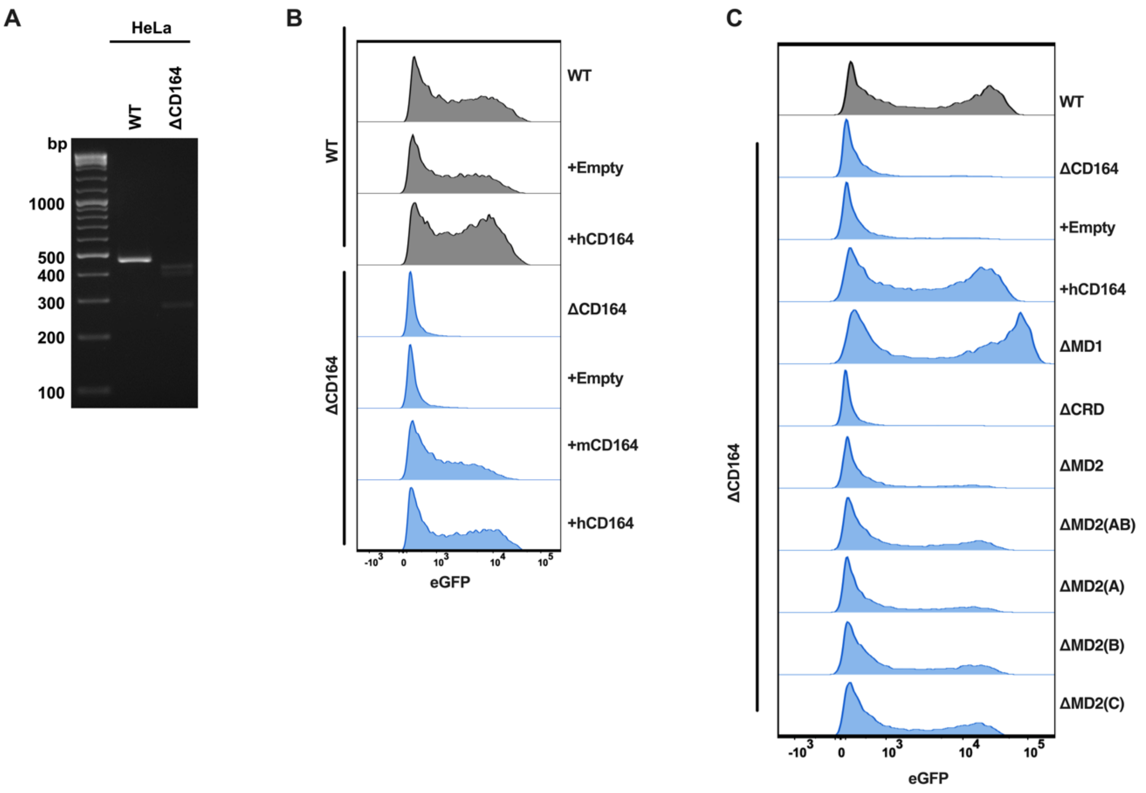
A
HeLa
WT
ΔCD164
bp
1000
500
400
300
200
100
B
WT
ΔCD164
WT
+Empty
+hCD164
ΔCD164
+Empty
+mCD164
+hCD164
eGFP
C
WT
ΔCD164
ΔCD164
+Empty
+hCD164
ΔMD1
ΔCRD
ΔMD2
ΔMD2(AB)
ΔMD2(A)
ΔMD2(B)
ΔMD2(C)
eGFP

**Supplemental Figure 3. Related to Figure 3.**

A) Qualitative PCR on parental HeLa and CD164 KO cells. By combining two gRNA’s an exon-intron boundary is removed, as confirmed by the smaller band size.

B) Representative flow cytometry data from Figure 3A.

C) Representative flow cytometry data from Figure 3C.

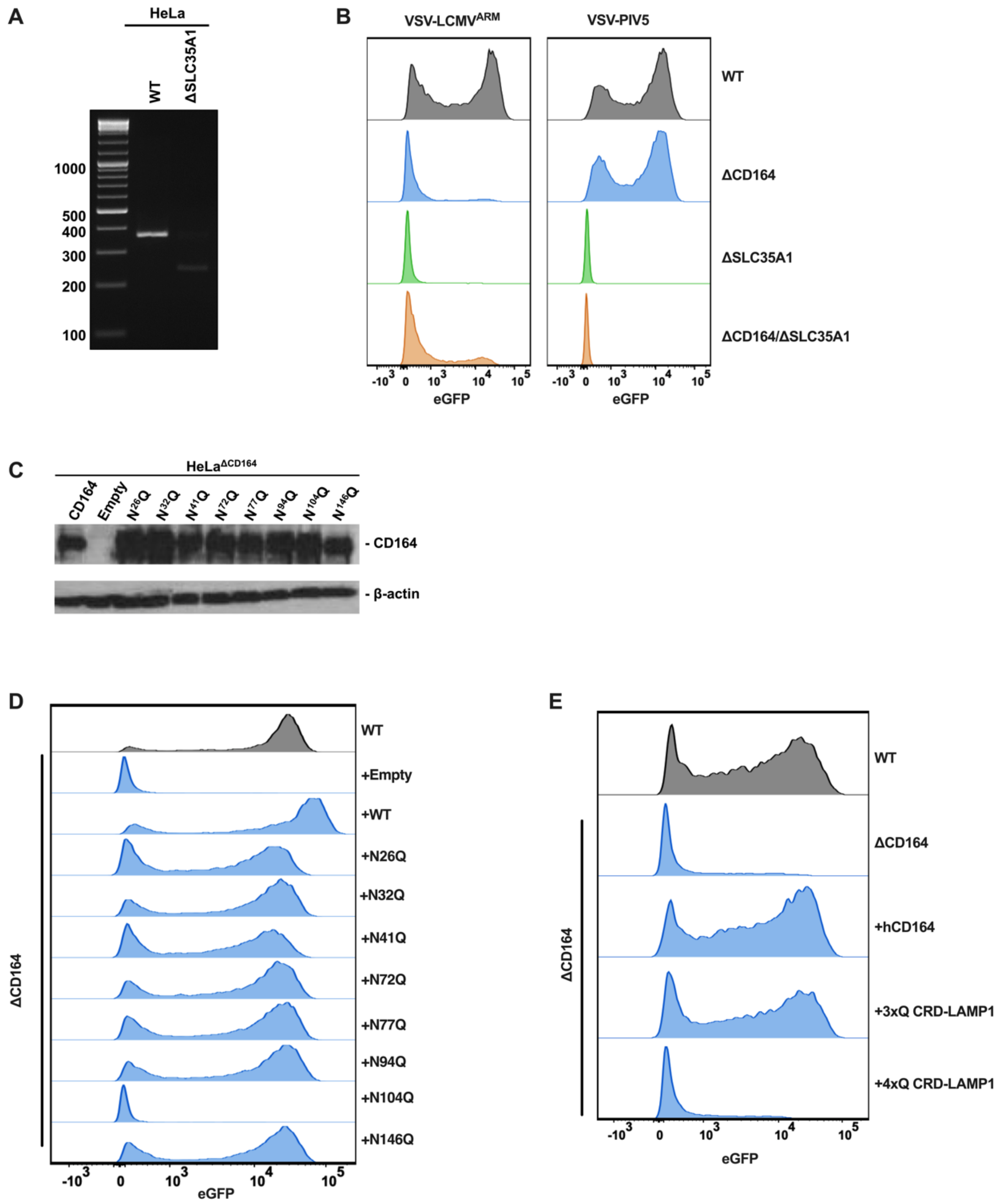
A
HeLa
WT
ΔSLC35A1
1000
500
400
300
200
100
B
VSV-LCMV^ARM
VSV-PIV5
WT
ΔCD164
ΔSLC35A1
ΔCD164/ΔSLC35A1
eGFP
eGFP
C
HeLa^ΔCD164
CD164
Empty
N26Q
N32Q
N41Q
N72Q
N77Q
N94Q
N104Q
N146Q
- CD164
- β-actin
D
WT
+Empty
+WT
+N26Q
+N32Q
+N41Q
+N72Q
+N77Q
+N94Q
+N104Q
+N146Q
ΔCD164
eGFP
E
WT
ΔCD164
+hCD164
+3xQ CRD-LAMP1
+4xQ CRD-LAMP1
ΔCD164
eGFP

**Supplemental Figure 4. Related to Figure 4.**

A) Qualitative PCR on parental HeLa and SLC35A1 gene KO cells. By combining two gRNA's an exon-intron boundary is removed, as confirmed by the smaller band size.

B) Representative flow cytometry data from Figure 4A.

C) Immunoblot analysis of the different N-linked glycosylation mutants. Briefly, **Δ**CD164 Hela cells expressing the different CD164 N-linked glycan mutants were lysed and subjected to immunoblot analysis using an anti-human CD164 antibody.

D) Representative flow cytometry data from Figure 4C.

E) Representative flow cytometry data from Figure 4E.

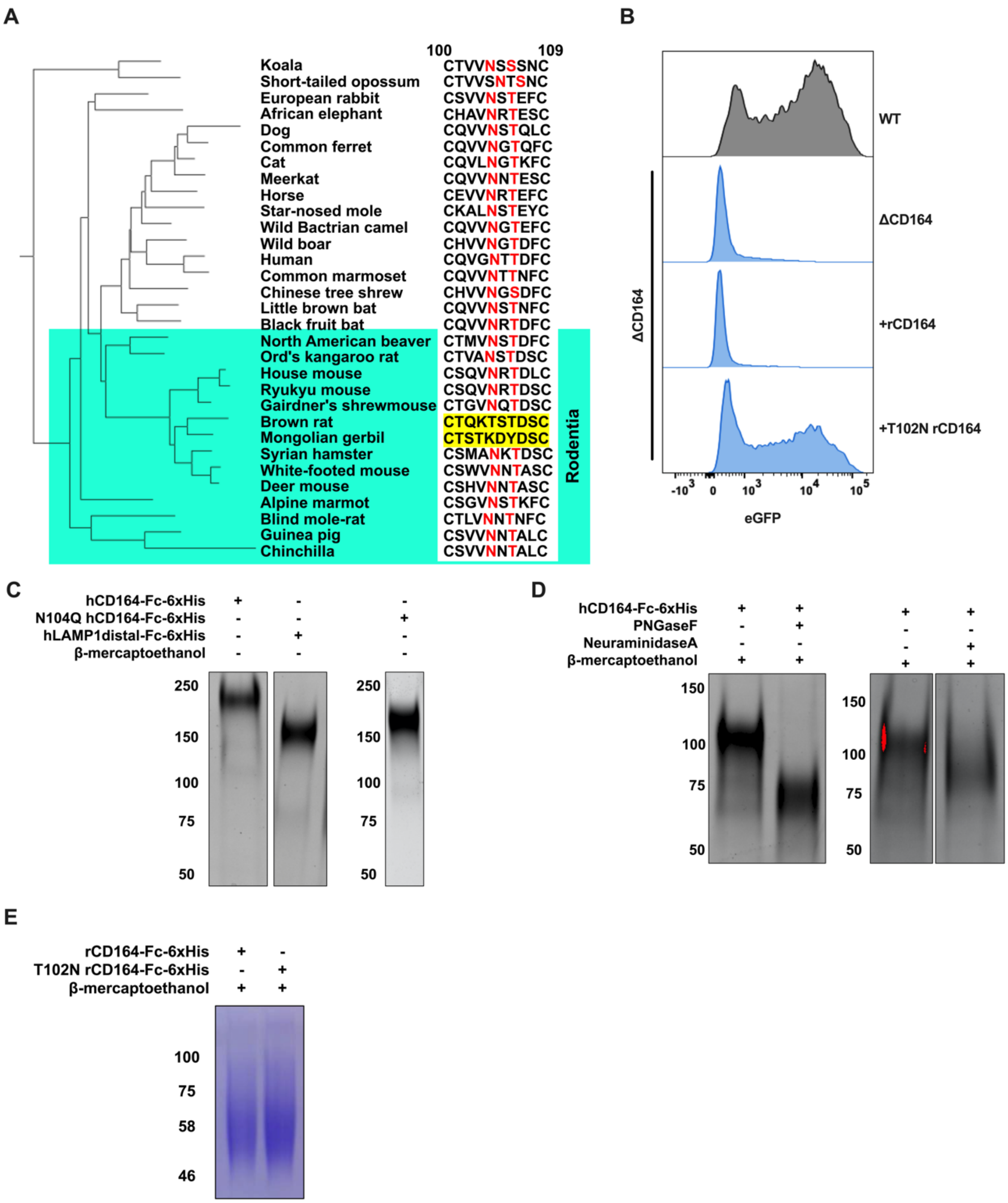
A
100
109
Koala CTVVNSSSNC
Short-tailed opossum CTVVSNTSNC
European rabbit CSVVNSTEFC
African elephant CHAVNRTESC
Dog CQVVNSTQLC
Common ferret CQVVNGTQFC
Cat CQVLNGTKFC
Meerkat CQVVNNTESC
Horse CEVVNRTEFC
Star-nosed mole CKALNSTEYC
Wild Bactrian camel CQVVNGTEFC
Wild boar CHVVNGTDFC
Human CQVGNTTDFC
Common marmoset CQVVNTTNFC
Chinese tree shrew CHVVNGSDFC
Little brown bat CQVVNSTNFC
Black fruit bat CQVVNRTDFC
North American beaver CTMVNSTDFC
Ord's kangaroo rat CTVANSTDSC
House mouse CSQVNRTDLC
Ryukyu mouse CSQVNRTDSC
Gairdner's shrewmouse CTGVNQTDSC
Brown rat CTQKTSTDSC
Mongolian gerbil CTSTKDYDSC
Syrian hamster CSMANKTDSC
White-footed mouse CSWVNNTASC
Deer mouse CSHVNNTASC
Alpine marmot CSGVNSTKFC
Blind mole-rat CTLVNNTNFC
Guinea pig CSVVNNTALC
Chinchilla CSVVNNTALC
Rodentia
B
WT
ΔCD164
+rCD164
+T102N rCD164
ΔCD164
-10^3 0 10^3 10^4 10^5
eGFP
C
hCD164-Fc-6xHis + - -
N104Q hCD164-Fc-6xHis - - +
hLAMP1distal-Fc-6xHis - + -
β-mercaptoethanol - - -
250 150 100 75 50
250 150 100 75 50
D
hCD164-Fc-6xHis + + + +
PNGaseF - + - -
NeuraminidaseA - - - +
β-mercaptoethanol + + + +
150 100 75 50
150 100 75 50
E
rCD164-Fc-6xHis + -
T102N rCD164-Fc-6xHis - +
β-mercaptoethanol + +
100 75 58 46

**Supplemental Figure 5. Related to Figures 4 and 5.**

A) Phylogenetic tree based on the amino acid sequence of CD164 from different mammals using MEGA sequence alignment software. The alignment shows the region surrounding the N104 loci with the sequon denoted in red. The region surrounding the N104 loci of Brown rat and Mongolian gerbil CD164 is highlighted in yellow.

B) Representative flow cytometry data from Figure 4F.

C) Soluble recombinant hCD164-Fc, N104Q hCD164-Fc, and LAMP1distal-Fc were incubated with non-reducing sample buffer and were run in an SDS-PAGE stain free gel (BioRad) and imaged using a gel scanner.

D) Soluble recombinant hCD164-Fc was treated overnight with PNGaseF or NeuraminidaseA. Sample was then buffer exchanged to TBS to stop the enzymatic reaction. Samples were run in a SDS-PAGE stain free gel (BioRad) under reducing conditions and imaged using a gel scanner.

**E)** Soluble recombinant rCD164-Fc and T102N rCD164-Fc were incubated with reducing sample buffer and were run in an SDS-PAGE gel. The gel was stained with Coomassie blue, destained, and imaged on a gel scanner.

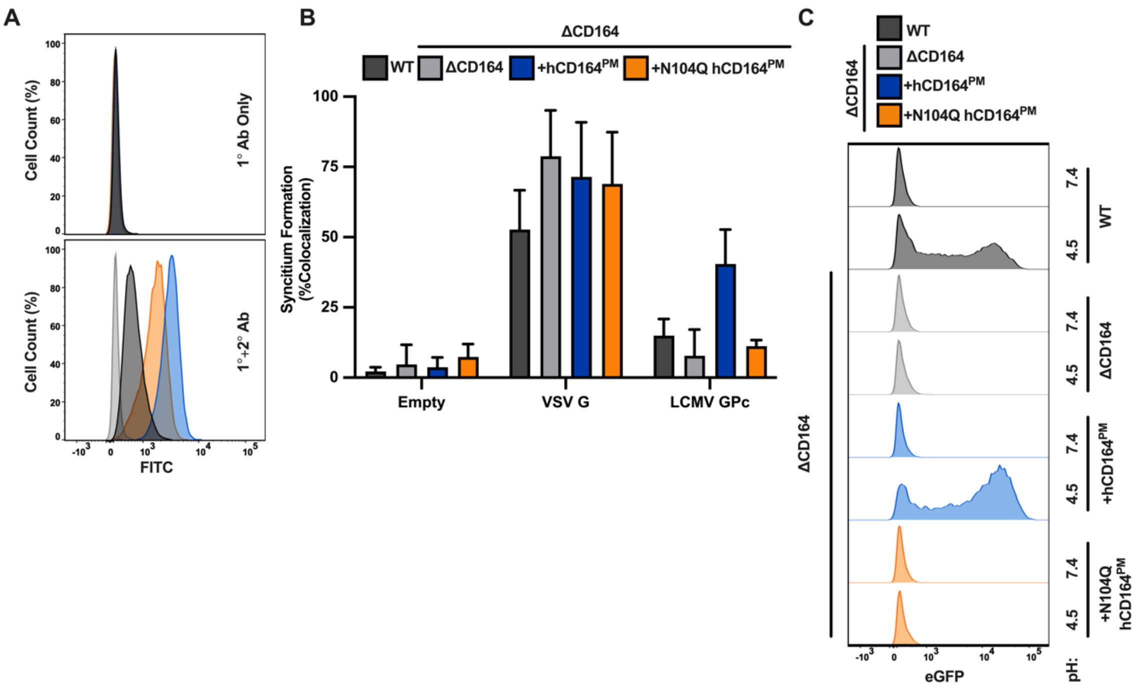
A
Cell Count (%)
1° Ab Only
1°+2° Ab
FITC
B
ΔCD164
WT
ΔCD164
+hCD164PM
+N104Q hCD164PM
Syncitium Formation (%Colocalization)
Empty
VSV G
LCMV GPc
C
WT
ΔCD164
+hCD164PM
+N104Q hCD164PM
eGFP
pH:
7.4
4.5

**Supplemental Figure 6. Related to Figure 6.**

A) WT, **Δ**CD164, and **Δ**CD164 expressing plasma-membrane localized CD164 mutants (hCD164$^{PM}$ and N104Q hCD164$^{PM}$) were cell-surface stained with an anti-CD164 antibody and were subjected to flow cytometry analysis to determine the level of cell-surface expression of CD164.

B) Quantification of syncytia formation from Figure 6A, with VSV G and Empty vectors included as positive and negative controls, respectively. Bar plot showing the average syncytia formation between three independent experiments. The fraction of formed syncytia was automatically calculated as surface area covered by syncytia post acidification divided by the surface area covered by cells prior to acidification.

C) Representative flow cytometry data from Figure 6B.

A

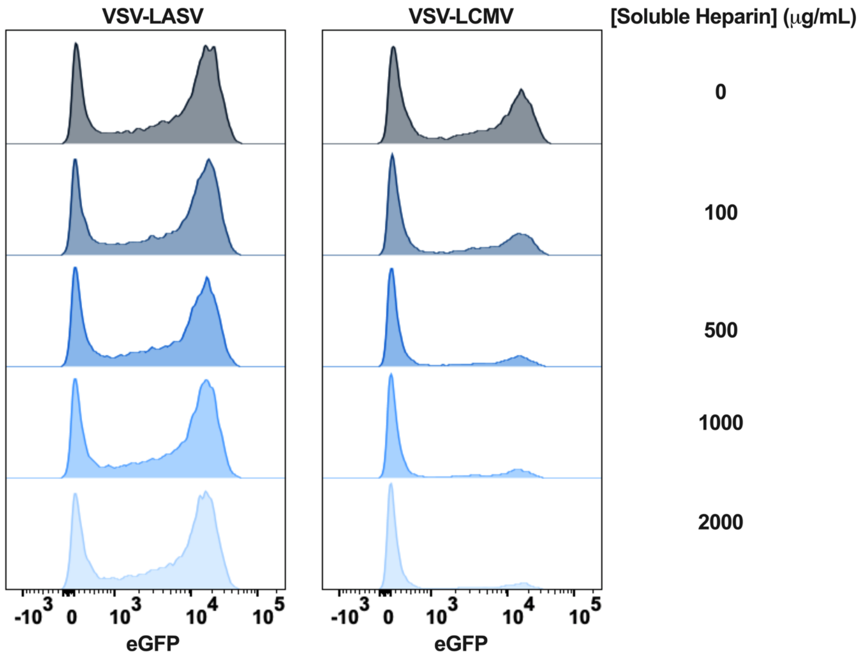

VSV-LASV
VSV-LCMV
[Soluble Heparin] (μg/mL)
0
100
500
1000
2000
-10^3 0 10^3 10^4 10^5
eGFP
-10^3 0 10^3 10^4 10^5
eGFP

**Supplemental Figure 7. Related to Figure 7.**

A) Representative flow cytometry data from Figure 7B.
